# Diagnostic Power of Circulatory Metabolic Biomarkers as Metabolic Syndrome Risk Predictors in Community-Dwelling Older Adults in Northwest of England (A Feasibility Study)

**DOI:** 10.3390/nu13072275

**Published:** 2021-06-30

**Authors:** Razieh Hassannejad, Hamsa Sharrouf, Fahimeh Haghighatdoost, Ben Kirk, Farzad Amirabdollahian

**Affiliations:** 1 Isfahan Cardiovascular Research Center, Cardiovascular Research Institute, Isfahan University of Medical Sciences, Isfahan 8158388994, Iran; razieh.hassannejad@gmail.com (R.H.); f_haghighatdoost@yahoo.com (F.H.); 2 School of Health Sciences, Liverpool Hope University, Liverpool L16 9JD, UK; 17007418@hope.ac.uk (H.S.); ben.kirk@unimelb.edu.au (B.K.); 3 Department of Medicine-Western Health, Melbourne Medical School, The University of Melbourne, Furlong Road, St. Albans, Melbourne, VIC 3021, Australia; 4 Australian Institute for Musculoskeletal Science (AIMSS), Geroscience & Osteosarcopenia Research Program, The University of Melbourne and Western Health, St. Albans, Melbourne, VIC 3201, Australia

**Keywords:** metabolic syndrome, biomarkers, association, older adults, insulin

## Abstract

Background: Metabolic Syndrome (MetS) is a cluster of risk factors for diabetes and cardiovascular diseases with pathophysiology strongly linked to aging. A range of circulatory metabolic biomarkers such as inflammatory adipokines have been associated with MetS; however, the diagnostic power of these markers as MetS risk correlates in elderly has yet to be elucidated. This cross-sectional study investigated the diagnostic power of circulatory metabolic biomarkers as MetS risk correlates in older adults. Methods: Hundred community dwelling older adults (mean age: 68.7 years) were recruited in a study, where their blood pressure, body composition and Pulse Wave Velocity (PWV) were measured; and their fasting capillary and venous blood were collected. The components of the MetS; and the serum concentrations of Interleukin-6 (IL-6), Tumor Necrosis Factor-α (TNF-α), Plasminogen Activator Inhibitor-I (PAI-I), Leptin, Adiponectin, Resistin, Cystatin-C, C-Reactive Protein (CRP), insulin and ferritin were measured within the laboratory, and the HOMA1-IR and Atherogenic Index of Plasma (AIP) were calculated. Results: Apart from other markers which were related with some cardiometabolic (CM) risk, after Bonferroni correction insulin had significant association with all components of Mets and AIP. These associations also remained significant in multivariate regression. The multivariate odds ratio (OR with 95% confidence interval (CI)) showed a statistically significant association between IL-6 (OR: 1.32 (1.06–1.64)), TNF-α (OR: 1.37 (1.02–1.84)), Resistin (OR: 1.27 (1.04–1.54)) and CRP (OR: 1.29 (1.09–1.54)) with MetS risk; however, these associations were not found when the model was adjusted for age, dietary intake and adiposity. In unadjusted models, insulin was consistently statistically associated with at least two CM risk factors (OR: 1.33 (1.16–1.53)) and MetS risk (OR: 1.24 (1.12–1.37)) and in adjusted models it was found to be associated with at least two CM risk factors and MetS risk (OR: 1.87 (1.24–2.83) and OR: 1.25 (1.09–1.43)) respectively. Area under curve (AUC) for receiver operating characteristics (ROC) demonstrated a good discriminatory diagnostics power of insulin with AUC: 0.775 (0.683–0.866) and 0.785 by cross validation and bootstrapping samples for at least two CM risk factors and AUC: 0.773 (0.653–0.893) and 0.783 by cross validation and bootstrapping samples for MetS risk. This was superior to all other AUC reported from the ROC analysis of other biomarkers. Area under precision-recall curve for insulin was also superior to all other markers (0.839 and 0.586 for at least two CM risk factors and MetS, respectively). Conclusion: Fasting serum insulin concentration was statistically linked with MetS and its risk, and this link is stronger than all other biomarkers. Our ROC analysis confirmed the discriminatory diagnostic power of insulin as CM and MetS risk correlate in older adults.

## 1. Introduction

Aging is portrayed by a cumulative deterioration in physiological functions and metabolic operations which result in morbidity and mortality [1]. The aging process is characterized by Insulin Resistance (IR), alterations in body composition and hormonal reductions, for instance, in insulin-like growth factor-1, growth hormone and sex steroids [2]. Reduction in sex hormones in elderly individuals (i.e., testosterone in men and progesterone in women at menopause) is a significant factor in the accumulation of abdominal adiposity [3]. It has been shown by computational tomography scans that aging is linked with reduction in subcutaneous fat and increase in visceral fat, which is also related to IR and the development of coronary heart disease [4]. Abdominal adiposity, in turn, plays a key role in the development of metabolic syndrome (MetS) [5].

### 1.1. Metabolic Syndrome

The most common definitions of MetS are produced by World Health Organization (WHO), National Cholesterol Education Program (NCEP)-Adult Treatment Panel III (ATP III), International Diabetes Foundation (IDF), American Heart Association (AHA) and the European Group for Study of Insulin Resistance (EGIR) (Table 1). These definitions generally refer to a cluster of metabolic conditions including clinical symptoms and abnormal laboratory results linked to abdominal obesity, hypertension, impaired glucose tolerance, IR, elevated triglycerides (TGs) and low level of high-density lipoprotein cholesterol (HDL-C) concentration [5,6]. These metabolic abnormalities present significant risk factors for cardiovascular disease (CVD) and diabetes [6]. Similarly, the term ‘Cardiometabolic Risk’ (CM) is portrayed by the occurrence of the components of MetS, specifically, abdominal adiposity, impaired glucose metabolism, hypertension and dyslipidemia [7,8,9]. 

### 1.2. Prevalence of Age-Related Metabolic Syndrome

The prevalence of MetS is increasing in accordance with increasing obesity rates as well as age [11]. Tune et al. [12] reported that ~20% of adults in the Western world are affected by MetS. Kaur et al. [13] estimated the worldwide prevalence of MetS to be between 10 and 48% depending on the age, gender, ethnicity and race of the population, while the IDF approximates that 25% of the world’s population has MetS [11,13]. Although the MetS is found to be linked with age, the mechanism of this dysfunction amongst elderly is still understudied [14]. Several studies have undertaken to determine the aging-specific prevalence of MetS within specific countries and populations [11]. In the UK, 1 out of 3 older adults aged 50 years and over had MetS [15]; in the USA, it is estimated that 43.5% of elderly have MetS [16]. Moreover, based on the American Heart Association (AHA) data, 50% of adults aged 60 years and over have MetS [17]. 

In 2015, it was reported that more than 1 in 4 adults in England (31% of men; 26% of women) are affected by hypertension, which contributed to 75,000 deaths [18]. Half of all people with diabetes in the UK are aged over 65 years and a quarter are aged over 75 [19] and, by 2025, it is estimated that more than four million people will live with diabetes in the UK [20]. Furthermore, it is estimated that more than 27% of all mortality rates in the UK are linked to heart and circulatory diseases [21]. Therefore, it is imperative to investigate early diagnostic perspectives and to decrease complications of the MetS amongst elderly.

### 1.3. Biomarkers Associated with Age-Related Metabolic Syndrome

Aging is found to be related to an increase in proinflammatory adipokines or cytokines, which impacts insulin action [22]. Cytokines are secreted by age-linked accumulated visceral adipose tissue as well as by the increase in age-associated senescent cells production [22]. The accumulation of adipocytes is linked with the abnormal production of adipokines that plays a key role in the development of the MetS [23]. The mechanism behind the association between adipose accumulation and the homeostatic dysregulation resulting in MetS has yet to become fully elucidated; however, obesity induced systemic oxidative stress has been mentioned, during which mitochondrial and peroxisomal oxidation of fatty acids can produce reactive oxidative species in oxidative reactions at cellular levels. Several lipid peroxidation end products (e.g., Malondialdehyde) are shown to be excessively produced in obesity and the IR of MetS and studies have demonstrated that such substances can enhance the expression of the pro-inflammatory cytokines, resulting in systemic stress [6]. Within the next few paragraphs, we firstly introduce some of the circulatory biomarkers associated with age-related MetS and then we will introduce the aim of the current study. More biomarkers of metabolic syndrome with biochemical background and clinical significance can be consulted by the interested reader in a previously published review paper [24].

#### 1.3.1. Interleukin-6 (IL-6)

IL-6 plays a key function in the natural inflammatory response. It is derived from M1 macrophages as part of the normal inflammatory reaction in response to infection and injury [25]. Both in vitro and in vivo studies confirmed that IL-6 can suppress lipoprotein lipase function [26]. Additionally, it is positively correlated with BMI, fasting insulin and the development of T2DM [13]. IL-6 is negatively linked to HDL-C levels [13]. In MetS, as a result of adipose dysfunction, M1 macrophage count increases inside the adipose tissue and thereby increases the release of IL-6 as well as other pro-inflammatory cytokines from adipose tissue [25]. IL-6 can operate in several cellular signaling pathways, such as mechanistic Target of Rapamycin (mTOR) and Protein Kinase C (PKC), to stimulate IR [6]. IL-6 was found to be linked to vascular dysfunction and atherosclerosis via its inflammatory features. Moreover, IL-6 can result in abnormal insulin signaling cascades, unusual Insulin function and impairment of glucose metabolism [25]. Elevated levels of circulatory IL-6 are shown to be associated with each of the MetS components [27,28,29]. Chedraui et al. has found that elevated levels of circulatory IL-6 in postmenopausal females were linked to abdominal obesity, low HDL-C and high TGs [28]. Therefore, adding IL-6 to the diagnosis panel of MetS should be justified [6].

#### 1.3.2. Insulin

Insulin is an anabolic hormone and its core function is to decrease the amount of glucose in the blood by promoting carbohydrate metabolism and storage as glycogen inside muscle and liver cells [30]. Insulin also promotes fat storage in adipose tissue as well as the cellular uptake of amino acids and assembly of those amino acids into proteins [31]. Furthermore, IR is a pathophysiological condition whereby a normal insulin concentration does not adequately promote a normal insulin response in the peripheral target tissues such as adipose, muscle and liver [13], which results in hyperinsulinemia. This can result in the overexpression of insulin activity in some normally sensitive tissues that results in the clinical manifestation of MetS [13]. In fact, IR, which is common among older adults, is a remarkable contributor in the development of MetS due to its role in impairing gluconeogenesis, lipogenesis, defective glycogen synthesis and glucose uptake in skeletal muscle [2]. Furthermore, abdominal adiposity, which is linked with aging, is obviously associated with IR due to its role in reducing insulin-mediated glucose uptake [32].

#### 1.3.3. Tumor Necrosis Factor-Alpha (TNF-α)

Tumor Necrosis Factor-Alpha (TNF-α) is a pro-inflammatory cytokine secreted via visceral adipose tissue [33]. It plays a key role in lipid metabolism and insulin signaling and thereby obesity. High levels of TNF-α were found to inhibit the production of Adiponectin [34]. It has been suggested that TNF-α promotes IR by the suppression of Insulin receptor substrate 1 signaling pathway [26]. On the other hand, the elevated TNF-α levels also promote abdominal obesity via the activation of mTOR and PKC signaling pathways [6]. There is a positive correlation between plasma TNF-α and each of body weight, WC and TGs, while TNF-α levels can negatively affect HDL-C levels. This explains the link between elevated levels of circulatory TNF-α and MetS [13]. A study of middle-aged adults with MetS revealed that the elevated levels of serum TNF-α were linked to IR and high TGs. The TNF-α, IL-6 and leptin levels in these participants were higher than those in the control group, indicating the correlation between those cytokines and MetS progression [35]. Another study stated that circulatory TNF-α levels in patients with MetS were significantly higher than those in the control group [36]. Consequently, TNF-α may be a significant factor in the onset and development in MetS and its related diseases [6]. 

#### 1.3.4. Adiponectin

Adiponectin is a protective adipokine derived exclusively from adipose tissue and its levels are reduced in obesity, T2DM and MetS [5]. Adiponectin is reported to have anti-diabetic features as well as anti-atherogenic and anti-inflammatory effects [37]. Adiponectin operates as an endogenous insulin sensitizer via enhancing glucose uptake by increasing fatty acid oxidation and decreasing glucose synthesis by the liver [38]. However, low circulatory levels of adiponectin are independent risk factors for type 2 diabetes [39]. Furthermore, adiponectin was indicated as a MetS biomarker and hypoadiponectinemia was found to be linked to obesity [38] as well as to IR, hyperinsulinemia and the development of T2DM independent of fat mass [26]. Adiponectin is inversely correlated with CVD risk factors comprising BP, LDL-C and TGs due to its role in vasodilation and repressing hepatic gluconeogenic enzymes as well as the production of endogenous glucose in the liver [13]. This elucidates the negative relationship between adiponectin and MetS. It has been documented that plasma adiponectin levels are inversely linked to the mean number of components of MetS in female and male Japanese adults, with higher Adiponectin levels found in females than in males; this is postulated as one of the potential mechanisms for explaining why females have a lower risk of coronary artery disease than males [23]. Several studies reported a negative relationship between serum adiponectin and the number of components of MetS independent of BMI [40,41,42]. It should be noted that the high molecular weight (HMW) form of adiponectin was suggested to be the most reliable biomarker in MetS diagnosis [43] and, therefore, adiponectin to be preferably considered on the MetS diagnosis panel of biomarkers. 

#### 1.3.5. Leptin

Leptin is an adipokine which is produced mainly by adipocytes but also via vascular smooth muscle cells, cardiomyocytes and placenta in pregnant females [6]. Leptin was found to trigger satiety and decrease energy intake plus increase energy expenditure. It also plays a key function in glucose synthesis and ameliorates insulin sensitivity [38]. Elevated levels of serum leptin were found to be correlated with MetS in many populations. This is elucidated as leptin is linked with obesity, IR, myocardial infarction and congestive heart disease [44]. Moreover, elevated leptin levels were demonstrated to be associated with central adiposity among postmenopausal females [45], whereas another study in nondiabetic Lebanese males aged over 50 years has shown a strong correlation between elevated leptin levels and WC, but a weak correlation with the lipid profile which disappeared with BMI adjustment [41]. Moreover, Martins et al. found a positive correlation between elevated serum leptin and obesity, IR and hyperinsulinemia, but a weak correlation with the other components of MetS [46]. By contrast, a study in a Korean population has concluded that reduction in leptin levels may be protective against MetS regardless of weight status since serum leptin was found to rise as components of MetS increased regardless of weight loss [47]. Although findings from literature about the link between leptin and MetS are controversial, the general consensus is that the serum leptin is found to be elevated in individuals with MetS independent of BMI and, thus, can serve as an effective biomarker in MetS screening panel [6]. 

#### 1.3.6. Plasminogen Activator Inhibitor-I (PAI-I)

Plasminogen Activator Inhibitor-I (PAI-I) is a molecule that functions to modulate extracellular matrix remodeling and fibrinolysis [48]. Under physiologic conditions, PAI-I is secreted into the circulation or extracellular space from intra-abdominal adipocytes, platelets and the vascular endothelium or hepatocytes [49]. In individuals with abdominal obesity, plasma PAI-I levels are increase and is thereby linked to greater risks of intravascular thrombus and adverse cardiovascular outcomes such as myocardial infarction and deep vein thrombosis [50,51]. In MetS, elevated levels of PAI-I exist; however, the link between PAI-I and the components of MetS is controversial. While PAI-I circulatory levels are found to be strongly correlated with BMI, TGs and IR [52], PAI-I levels are found not to be linked with dyslipidemia, but rather with the distribution phenotype of adipocytes, i.e., visceral adipose tissue and ectopic fat in the liver [53,54]. It was also found that PAI-I levels are gender-dependent (higher in males than females) [54]. PAI-I levels are reported to decrease with calorie restriction, weight loss, fat mass reduction and when IR improves [32,48]. 

#### 1.3.7. Resistin

Resistin is a peptide hormone that performs several functions in metabolism and possesses physiological roles linked to inflammation [55], endothelial dysfunction [56], cardiomyocyte function [57], as well as cholesterol metabolism [58]. Literature about the role of Resistin in insulin sensitivity and obesity is controversial due to the difference between human and rodent resistin [59,60]. The ability of resistin to modulate glucose metabolism is related to the activation of the suppressor of cytokine signaling 3 (SOCS3), which is an inhibitor of insulin signaling in adipocytes [61,62]. Plasma resistin levels were found to be correlated with markers of inflammation, such as IL-6 and TNF-α, and thus could predict coronary atherosclerosis in humans independent of CRP. Therefore, resistin may represent a novel link between metabolic signals, inflammation and atherosclerosis [63,64]. Zahary et al. [58] reported that serum resistin is positively correlated with few CM risk factors such as BMI, WC, FBG and total cholesterol. Moreover, the Multi-Ethnic Study of Atherosclerosis (MESA), which included 1913 participants from different ethnicities in the USA, concluded that there was a strong correlation between elevated circulatory resistin and the increased incidence of heart failure, all CVD events independent of traditional cardiovascular risk factors, obesity, markers of inflammation/IR and several adipokines [65]. Furthermore, resistin is found to be associated with obesity, visceral fat, inflammation and IR and it can be a potential risk factor of MetS and comorbidities of CVD [66]. However, genetic determinants of this adipokine may provide potential clues about its role in human susceptibility of disease [59]. 

#### 1.3.8. C-Reactive Protein (CRP)

C-Reactive Protein (CRP) is an acute-phase protein biomarker that is generated in response to acute injury, infection or other inflammatory stimuli [31]. As a clinical marker of inflammation, elevated levels of serum CRP are strong independent predictors of CVD in asymptomatic individuals [67]. Elevated levels of CRP are found to be linked with abdominal adiposity [68], IR [69], BMI [70] and hyperglycemia [68] and thereby it contributes to MetS onset. In fact, regardless of the presence of MetS in an individual, CRP levels can independently predict the future CVD [71]. Consequently, CRP may be a reliable biomarker in the diagnosis of MetS [13]. It should be noted that increasing levels of IL-6 during inflammation are linked to elevated levels of CRP [72], with IL-6 inducing the CRP gene [72].

#### 1.3.9. Ferritin

Ferritin is an ubiquitous intracellular protein that regulates iron homeostasis and considered as a biomarker in the assessment of iron stores in the human body [73]. Increasing evidence indicates that elevated body iron stores may be linked to adverse health outcomes such as type 2 diabetes mellitus [74,75]. In the Nurses’ Health Study, the participants in the highest quintile had a 2.5-fold higher diabetes risk than those in the lowest quintile [76]. Similarly, results from the cohort of the European Prospective Investigation into Cancer and Nutrition study (EPIC) have also shown that higher iron stores below the level of hemochromatosis are linked with increased risk of type 2 diabetes [77]. Moreover, elevated serum ferritin levels were shown to be associated with CVD risk in several studies [78,79,80]. However, the mechanisms for the association between elevated circulatory ferritin levels and MetS components have yet still to be determined [81]. A meta-analysis of 14 studies (56,053 participants) by Abril-Ulloa et al. [73] found that elevated ferritin levels are independently and positively associated with the presence of MetS with an odds ratio higher than 1.73. The results of this meta-analysis suggested that MetS could already develop in males and in premenopausal females at ferritin levels that are lower than the WHO cut-offs for iron overload [73]. A systematic review and meta-analysis of 26 studies by Suarez-Ortegon et al. [81] have shown that elevated serum ferritin was strongly associated with elevated triglycerides and glucose levels. They also found that hepatic injury, BMI and type of ferritin assay appear to affect the association between ferritin and MetS [81]. Based on evidence, there is a bi-directional interrelation between glucose and iron metabolism [82,83]. Moreover, elevated ferritin levels have been found to be associated with other components of MetS including hypertension [84], dyslipidemia [78,85], elevated fasting insulin and blood glucose levels [86] and central adiposity [87]. However, more prospective studies are needed to investigate the validity of high serum ferritin as a biomarker of MetS risk and diagnosis [73].

#### 1.3.10. Cystatin-C

Cystatin-C is a low molecular weight alkaline protein and it is broadly distributed and found in most bodily fluids [88]. It has been reported that cystatin-C is an independent and strong predictor of cardiovascular events, diabetes and all-cause mortality [89,90,91]. Cystatin-C is also found to be closely associated with the inflammation [92] as well as inflammatory biomarkers comprised of CRP, IL-6, TNF-α and others [93]. As a key marker of renal function, cystatin-C may be closely related to MetS whereby patients carry high risk for CVD as well as renal dysfunction [89,91,94]. It has been reported by Liu et al., that the higher the MetS component scores, the higher the serum cystatin-C concentration in MetS patients [16]. However, the mechanism of cystatin-C’s association with MetS is still unclear. It has been reported that cystatin-C concentrations are significantly correlated with IR [95] and, moreover, that each component of the MetS may results in renal dysfunction [91]. Renal function impairment is linked to elevated serum cystatin-C levels in MetS patients [95]. It has been shown that cystatin-C can act as a fat-derived secretory adipocytokine [96] and that it can thereby affect the functions of adipose tissue and induces obesity-linked complications [97]. Finally, cystatin-C may induce, be a risk factor or cause of MetS by an oxidative stress mechanism [98], which is the question to be elucidated. In a cross sectional study of 925 dyslipidemic patients over 10 months, Servais et al. found that circulatory cystatin-C levels were significantly higher in MetS patients than in others (0.86 ± 0.23 vs. 0.79 ± 0.20 mg/L, respectively, *p* < 0.0001) [91]. Therefore, cystatin-C may potentially be a reliable marker of MetS.

### 1.4. Biomarkers Associated with Age-Related Metabolic Syndrome—Opportunities and Challenges

Within the medical and nutritional sciences, we use biomarkers to support the diagnosis and management of clinical conditions and nutritional deficiencies, especially in the absence of overt/adequate clinical signs and symptoms or as an element of the assessment of the nutritional status to complete dietary, anthropometrical and clinical assessments. Advance biomarkers can provide a tool for the quantification of susceptibility to disease and effective estimation of the risk within the population [6]. Similarly, potential advance MetS biomarkers can provide means for early detection and management of the syndrome and its subsequent complications amongst older patients and for risk stratification in aging populations. Within the recent years, modern and innovative clinical chemistry assays such as multiplex analysis via Luminex-100 [99] or Biochip Array Technology via Evidence Investigator [100] have been developed for the assessment and profiling of the advance biomarkers of MetS, confirming the potential and opportunities for assessment, diagnosis and early detection of the risk markers. 

Nevertheless, there are substantial challenges limiting the clinical and research use of the biomarkers in early detections and management of the MetS in older adults. Firstly, the laboratory facilities and the research expertise required for the assessment of these biomarkers are not widely available across the healthcare and clinical research settings. For instance, the enzyme-linked immunosorbent assay (ELISA), which is the most established clinical chemistry assay for the analysis of these markers, is expensive and requires laboratory expertise. The ELISA kits for the analysis of a single biomarker can cost USD 500–1000 and this is adequate for the analysis of 40 patient samples, while duplicate running of each patient samples is recommended for accurate assessment, limiting the wide use of the method as a cost-effective solution for the analysis of multiple biomarkers within large cohorts of participants and clinical trials [100]. The use of the multiplex analysis such as biochip array technology is also barely more affordable as it requires expensive research equipment and biochip (biochip immune-analyzer costing > USD 50,000 and biomarkers kits costing an average of USD 5000, which is adequate for the assessment of 43 patient samples and 9 calibrators [100]). Secondly, the direct association between the biomarkers and MetS and the potential predictive power of the individual circulatory markers in the early detection of the cardiometabolic risk have yet to be fully elucidated. We have earlier referred to the emerging but inconclusive literature on the roles of biomarkers in relation with MetS (please see points 1.3.1 to 1.3.10 discussed above) and there are several confounding factors (e.g., diet, exercise and ethnicity) potentially affecting the circulatory levels of these potential MetS biomarkers; this is more understandable when remembering that these circulatory biomarkers have multiple roles and complex roles and functions and are further involved in several physiological and metabolic pathways, producing a potential conceptual challenge for establishing the sensitivity and specificity of the individual marker for the MetS diagnostic purposes [6]. These biomarkers are not only associated with the pathophysiology of the MetS but also linked with each other as a moderator, mediator, synergist or inhibitor and, hence, the interlinking association between these markers especially in relation with MetS is understudied. Finally, in the absence of large-scale studies representing target populations (e.g., older adults), the diagnostic cut-off values of most of these markers for different age groups and populations have yet to be established [19] and the potential impact of aging on the association between these markers and MetS have been understudied. 

The inconsistencies, challenges and controversies on the reported findings of the link between biomarkers and MetS in the elderly demonstrate a gap in knowledge and a need to conduct further research in the field. Therefore, the aim of the current study was to examine the diagnostic power of circulatory metabolic biomarkers as Metabolic Syndrome risk correlates in community dwelling older adults in Northwest England.

## 2. Materials and Methods

### 2.1. Study Design

The study analyzes the cross-sectional baseline data collected as part of a randomized single-blind clinical trial (Registered within *Clinicaltrials.gov* under trial identifier NCT02912130) conducted in a single center within the Research laboratories of the School of Health Sciences, Liverpool Hope University in Liverpool, Merseyside, United Kingdom, between September 2016 and March 2018. The ethical approval for the Clinical Trial was granted from the Northwest of England NHS Research Ethics Committee UK (REC Number: 16/NW/0480) and the supplementary ethical approval for the re-analysis of the data was granted by the university Ethics committee. While the current research includes re-analysis of the secondary outcome measures of the trial (i.e., biomarkers of the cardiometabolic health); the additional information about the design, sample size calculation and primary and secondary outcomes measures of the trial can be found at: https://clinicaltrials.gov/ct2/home for registered clinical trial NCT02912130.

### 2.2. Participants

Participants were healthy community-dwelling older adults aged 60 years and over without severe pre-existing medical conditions. The sampling strategy included convenient and snowball approaches, with recruitment based on advertisement in local community centers and GP practice surgeries and shops, combined with the word of mouth and verbal face to face telephone or email communications. The target population was defined with the inclusion criteria of male and female older adults aged 60 to 90 years who reside in the northwest of England and able to speak and understand English and are willing to consent with respect to participation and to follow the study procedures. The exclusion criteria were set to remove participants with recent (up to three months) or concurrent participation in any other clinical trials or dietary and/or exercise intervention programs; participants with self-reported lactose intolerance or uncontrolled diabetes and hypertension; hypotension and/or psychological and mental illnesses; participants with a history of falls or history of osteoporosis; participants with undergoing medical, physical or hormonal therapies; patients with major clinical conditions and participants with medical conditions that precluded safe participation in the research. For further details on the definition of inclusion and exclusion criteria, please visit the link to the registered clinical trial provided in Section 2.1.

If eligible, participants were provided with a participants information sheet and an oral briefing about the procedures. In line with the ethical approval, all participants provided written informed consent prior to the commencement of the study. Eligible participants then completed several additional questionnaires about the demographics, nutritional status and the quality of life.

### 2.3. Procedures

After an overnight fasting from consuming any foods and drinks (other than water), participants attended the clinical laboratories of the School of Health Sciences at Liverpool Hope University for assessment of the outcome measures. The assessments of the outcome measures (e.g., body composition, pulse wave velocity (PWV), capillary and venous blood biomarkers) were conducted in the morning to minimize the impact of diurnal variation on health outcome measures. For further details about the procedures, please see our previous publications [101,102].

### 2.4. Outcome Measures

In the present study we used the NCEP-ATP III criteria (please see Table 1) for defining the MetS and its components.

#### 2.4.1. Body Composition and Blood Pressure

After removing shoes, socks, watches, jewelry and heavy clothing, participants’ height was determined to the nearest 0.1 cm in the standardized Frankfort Plane anatomical position using a SECA 213 stadiometer (SECA GMBH & Co., Hamburg, Germany). Body mass was measured to the nearest 0.1 kg by Tanita MC-180 MA (Tanita Ltd., Tokyo, Japan). Height and body mass were then used for the general calculation of BMI as commonly conducted within the practice. The circumferences of waist and hip were assessed using validated procedure advised in the literature [103] using a nontraceable tape measure over light clothing. Body composition, fat and muscle mass, total body water and the overall and segmental percentage body fat were assessed by a four terminal Maltron BioScan 920-II (Maltron International, Essex, UK), which is a multifrequency bioelectrical impedance analyzer (previously cross-validated with MRI amongst older adults [104]), while in a fasted state and supine position placed on a medical bed. Blood pressure was conducted as part of the measurement of the PWV. In order to perform this, a non-invasive pulse wave analysis was conducted to examine central aortic pressure waveform parameters and carotid-femoral pulse wave velocity (cf-PWV) using SphygmoCore XCEL arterial testing device (ATCOR Medical Ltd., New South Wales, Australia). For further details on this procedure, please see the measurement procedure within our earlier publication [105].

#### 2.4.2. Biomarkers

The full procedures for capillary and venous blood collection and analysis of biomarkers were detailed elsewhere [7,106]. In brief, after fasting overnight, 1.5 µL capillary whole blood was collected using a lancet, capillary tube/plunger and an assessment kit and then transferred to the Affinion AS100 blood analyzer (Abbott Laboratories, Chicago, IL, USA) for the assessment of glycated hemoglobin (HbA1C). A second sample of 35 µL was then collected and injected into the equipment cassette of Alere LDX analyzer (Alere, San Diego, CA, USA) to assess total cholesterol, high-density lipoprotein cholesterol (HDL-C) and triglycerides (TGs), while these variables were used by the analyzer for the calculation of low-density lipoprotein cholesterol (LDL-C). The Atherogenic index of plasma (AIP) was calculated as a logarithmically transformed ratio of molar concentrations of TGs to HDL-C [107].

Venous blood samples were collected from fasted participants with a 22 G (or if necessary smaller 23 G) needle and drawn into a 10 mL vacutainer tube (BD Diagnostics, Franklin Lakes, NJ, USA). Serum was then separated via centrifugation at 1300 rpm for 10 min at 4 °C and distributed into six aliquots of 250 µL for each participant and stored at −70 °C until used. A biochip array protein analyzer Evidence Investigator and Metabolic Syndrome Arrays I and II were used to measure cardiometabolic biomarkers using chemiluminescent multiplex immunoassays (Randox Laboratories, Antrim, UK). Metabolic Syndrome Array I was used to measure Interleukin-6 (IL-6), Tumor Necrosis Factor-α (TNF-α), insulin, leptin, ferritin, resistin and Plasminogen Activator Inhibitor-1 (PAI-1), while the Metabolic Syndrome Array II was used to examine adiponectin, C-reactive Protein (CRP) and cystatin-C, following the procedures instructed by the manufacturer. Further details of the assays are reported elsewhere [100,108,109,110,111]; however, we have outlined the details as follows. 

Frozen samples and reagents carriers containing protein chips were left at room temperature for 15 min in preparation for the assay. An assay diluent of 200 µL was added to each well of the chips using an Eppendorf pipette and then 100 µL of calibrator/control or serum samples was added. Ensuring that samples and diluent were mixed, the chips were placed in a thermo shaker at 37 °C at 370 rpm for an hour. Samples were then removed from the thermo shaker, sides tapped and liquid discarded. A quick wash was then conducted for all Metabolic Syndrome Array I and II, where the wells of chips were filled with wash solution, side tapped for 30 s and liquid discarded. After a more thorough washing of the chips with washing fluids for four and six repeats for Metabolic Syndrome Array I and Metabolic Syndrome Array II, respectively, 300 µL of conjugate fluids comprised of enzyme specific antibody was added to each well and the chip was placed on a thermo shaker for another hour at 37 °C and 370 rpm. Ten minutes before the completion of the incubation, working signal was prepared combining luminol-EV840 and peroxidase in a 1:1 ratio within an amber vial to avoid breakdown by light and a signal solution was gently swirled on a roller shaker for at least 5–10 min. When the incubation was complete, the wash steps were repeated (four to six times) as outlined above. When final wash was completed, a 250 µL of substrate reagent containing luminol and peroxidase was added to each well of the biochips and covered with foil to protect from light and after 2 min the carrier was placed in the Evidence Investigator. The chips signals were detected by a charged coupled device camera of the equipment and the images were then sent to the device computer for analysis in comparison with the calibration curve for each biomarker. 

### 2.5. Statistical Analysis

Data analysis was conducted using SPSS version 25 for Windows (IBM SPSS, Inc., Armonk, NY, USA). Descriptive statistics were conducted to establish the characteristics of the study populations, while inferential statistics were used to address the main research questions. Data were expressed as mean ± standard error (SE) or percent. In order to investigate the strength of the association between the biomarkers and body composition, regression analysis was used. The univariate and multivariate regression analyses were applied to evaluate the association of circulatory metabolic biomarkers with cardiometabolic risk parameters, while the Bonferroni method was used to adjust the type one error rate for multiple comparisons. The normality assumption of regression residual was examined graphically and by Kolmogorov–Smirnov test. In the case of a strong deviation of normality, log-transformation of outcome variables was used. In order to investigate the associate between CM risk factors (at least two risk factors and MetS) and biomarkers (namely IL-6, insulin, TNF-α, adiponectin, leptin, PAI-1, resistin, CRP, ferritin and cystatin-C), the odd-ratios and 95% confidence interval were calculated from logistic models in unadjusted and adjusted models via controlling for age, adiposity (i.e.,% body fat) and energy and macronutrients intake (i.e., total calorie intake and percentage contribution of energy from carbohydrates, fats and proteins) in the adjusted model. In order to examine the discriminatory power of each biomarkers to detect CM risk, the area under the receiver operating characteristics (ROC) curve (AUC) and area under the precision-recall curve (AUPRC) were quantified and examined considering the CM risk with at least two risk factors of MetS and the scenario including all three risk criteria of MetS. Cross-validation and bootstrapping were used to obtain unbiased estimates of predictive accuracy. All discriminatory power analysis was generated using the SAS software (SAS Institute Inc. 2013. SAS^®^ 9.4 Statements: Reference. Cary, NC, USA: SAS Institute Inc.) and by applying ROC, XVAL and BVAL macros [112].

## 3. Results

### 3.1. Participants’ Characteristics and Prevalence of Metabolic Syndrome

A total of 100 older aged adults participated in this study and the participants’ average age and BMI were 68.7 years and 27 kg/m^2^, respectively. The study population included 52% female and 97% were British (Table 2). 

### 3.2. Prevalence of Metabolic Syndrome

The prevalence of CM risk within the study population is summarized in Table 2. Using the definition of NCEP-ATP III, 26% of older adults were classified as affected by MetS, 97% had at least one risk factor and 59% had at least two CM risk factors. The most prevalent CM risk factor was hypertension (83.3% and 92.3% amongst men and women respectively). Amongst the biochemical markers included within the definition, elevated FBG was the most prevalent CM risk factor (38.7%) and reduced HDL-C was the lowest prevalent one (13.5%).

### 3.3. Association with Cardiometabolic Risk

**Figure 1:**
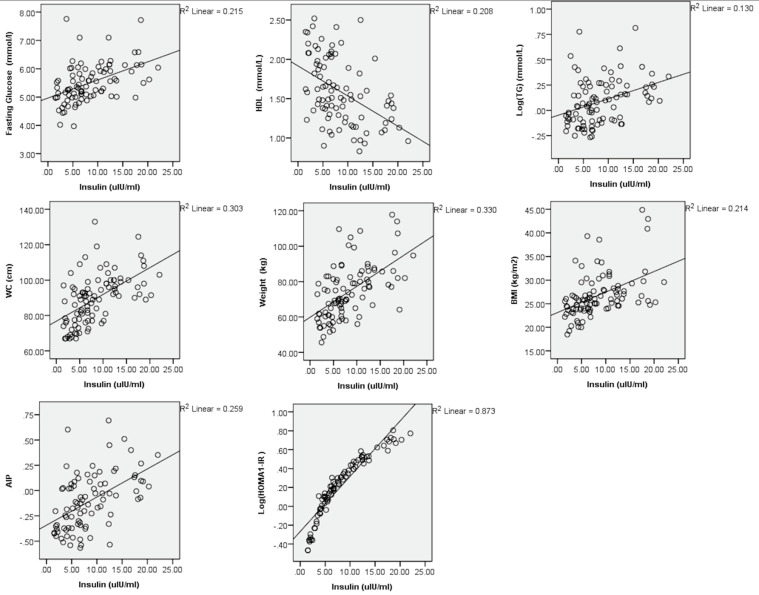
Linear regression of circulatory insulin with cardiometabolic risk presented to produce visual representation and examples for Table 3.

The univariate linear regression of circulatory metabolic biomarkers with cardiometabolic risk parameters is presented in Table 3. IL-6 was directly associated with BMI (r = 0.374) and percentage body fat (r = 0.386). Insulin was positively associated with FBG (r = 0.464), TGs (r = 0.361), WC (r = 0.550), BMI (r = 0.462), AIP (r = 0.509) and obviously HOMA1-IR (r = 0.934), while inversely associated with HDL-C (r = −0.457). These associations are also visually represented in Figure 1. On the contrary, adiponectin was inversely linked with WC (r = −0.396) and HOMA1-IR (r = −0.474) and directly linked with HDL-C (r = 0.294). Leptin was only statistically associated with BMI (r = 0.539) and percentage body fat (r = 0.632). PAI-I, resistin, ferritin and cystatin-C were not statistically associated with any cardiometabolic risk parameters, whereas CRP was only statistically associated with weight (r = 0.403), BMI (r = 0.526) and percentage body fat (r = 0.512). The multivariate linear regressions of circulatory metabolic biomarkers with cardiometabolic risk parameters are presented in Table 4, demonstrating that all anthropometric indices of adiposity, FBG, HDL-C, TGs, AIP and HOMA1-IR had statistically significant association with circulatory metabolic biomarkers.

The logistic regressions with unadjusted and multivariable adjusted odd ratio (OR) and 95% CI for examining the association between the biomarkers and the presence of at least two CM risk factor and at least three CM risk factors (i.e., diagnosed MetS) are demonstrated in Table 5. The adjusted model included the control of age, adiposity, energy and macronutrients intake as covariates. Insulin (OR = 1.33, 95% CI: 1.16–1.53), IL-6 (OR = 1.34, 95% CI: 1.03–1.74) and resistin (OR = 1.37, 95% CI: 1.06–1.78) were statistically associated with at least two CM risk factors and insulin (OR = 1.24, 95% CI: 1.12–1.37), IL-6 (OR = 1.32, 95% CI: 1.06–1.64), CRP (OR = 1.29, 95% CI: 1.09–1.54), TNF-α (OR = 1.37, 95% CI: 1.02–1.84) and resistin (OR = 1.27, 95%CI: 1.04–1.54) with the occurrence of the MetS; however, controlling for the confounding factors within the adjusted model eliminated these associations for all biomarkers except for insulin, which became stronger (OR = 1.87, 95% CI: 1.24–2.83 and OR = 1.25, 95% CI: 1.09–1.43 for at least two CM risk factors and the occurrence of the MetS, respectively). Interestingly, within our biomarker panel, PAI-I, leptin, adiponectin, cystatin-C and ferritin were not associated with the occurrence of CM risk factors altogether.

### 3.4. Discriminatory Diagnostic Power

**Figure 2:**
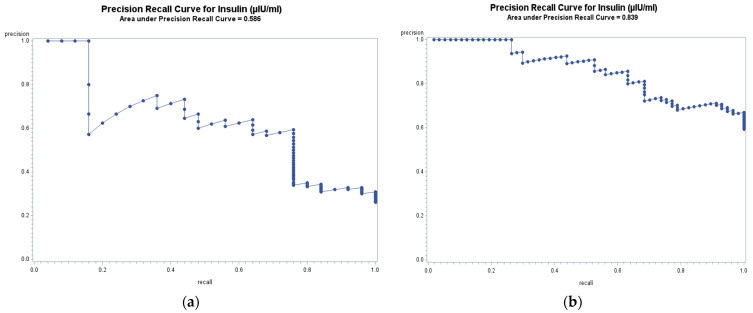
Precision-recall curve for insulin based on MetS as an outcome (**a**) and based on having at least two cardiometabolic risk factors as outcomes (**b**).

The ROC analysis investigating the area under curves and 95% confidence intervals of biomarkers for the assessment of discriminatory power in prediction of cardiometabolic risk are demonstrated in Table 6. In line with the results of the logistic regression, the highest AUC for MetS belonged to insulin, while the largest AUC for at least two CM risk factors also confirmed this finding. Figure 2 illustrates the precision-recall curves and area under precision recall curves (AUPRC) for insulin, showing consistency with AUC, and that insulin has the largest AUPRC for MetS and at least two CM risk factors (0.586 and 0.839, respectively). Full precision-recall curves based on MetS diagnosis and based on at least two cardiometabolic risk factors diagnosis for all markers can be seen within the Supplementary Figures S1 and S2. Diagnostic parameters in Table 7 also confirmed insulin’s diagnostic superiority since insulin has high precision and recall for MetS and for at least two CM risk factors resulting in higher AURPC. Balanced accuracy as an overall performance metric for the model was also the highest for insulin in all models. While the key diagnostic parameters with 5-fold cross validation are demonstrated in this table, a full versions of this information with bootstrap are presented within the Supplementary Table S1.

## 4. Discussion

To the best of our knowledge, this is the first study reporting the prevalence of CM risk and MetS in community-dwelling older adults in Liverpool and the first that compared the association between a variety of emerging circulatory metabolic biomarkers with measured body fat and CM risk in this population. The study addressed its research question about the clinical usefulness and diagnostic power of circulatory metabolic biomarkers as MetS risk correlates in older adults and contributed to our understanding of a broad picture of assessment of CM risk of older adults in the UK.

The present research revealed a significant and relatively strong association between circulatory insulin, adiposity and a range of CM risk indicators including WC, AIP, FBG, HDL-C and TGs and the inverse significant association between circulatory adiponectin, with WC and HOMA1-IR. Although the potential clinical uses of AIP as a blood lipid indicator associated with coronary artery disease, blood pressure, diabetes and vascular events have been recently reported in several other studies [107,113,114,115,116,117,118,119,120], to the best of our knowledge this is the first study examining its extensive association with range of CM biomarkers within this target population to establish its association with insulin. While pro-inflammatory IL-6 was surprisingly not associated with any blood biomarkers of MetS, there were statistically significant associations between IL-6 and the physical risk markers of MetS, namely with BMI and percentage body fat. Cystatin-C was the only biomarker that did not show any significant association with CM risk parameters, questioning the notion of whether it needs to be included within the diagnostic panel. With the exception of cystatin-C, HOMA1-IR was statistically and significantly associated with all potential circulatory metabolic biomarkers, confirming the potential association between these circulatory CM biomarkers and IR, which is previously reported within the literature [121,122]. The multivariate linear regression of circulatory metabolic biomarkers with cardiometabolic risk also demonstrated that all anthropometric indices of adiposity, FBG, HDL-C, TGs, AIP and HOMA1-IR had statistically significant association with circulatory metabolic biomarkers.

While logistic regression with the unadjusted model showed a potential for insulin, IL-6, resistin and possibly TNF-α as correlates of CM risk, when multivariate adjusted OR was conducted controlling for gender, adiposity, energy and macronutrient intake, only circulatory level of insulin remained as the independent predictor of the CM risk. The ROC examination also confirmed the clinical usefulness of insulin based on the consistent greatest AUC and AUPRC in detection of at least two CM risk factors and the three CM risk factors confirming its superior diagnostic power in the detection and prediction of MetS. A high AUPRC represents both high recall and high precision, where high precision relates to a low false positive rate and high recall relates to a low false negative rate. High scores for both show that the insulin is returning accurate results (high precision), as well as returning a majority of all positive results (high recall). In addition, the highest balanced accuracy shows good overall performance of all models. For IL-6, the potential association with cardiometabolic risk in logistic regression with unadjusted models that faded away in the adjusted model can be interpreted broadly in line with the literature, especially considering the association between IL-6 and measures of body composition such as BMI and percentage body fat reported in the current study. We therefore postulated that this association may be moderated by body composition, as is reported in animal models [123], although we could not confirm the other interrelations between IL-6, low level of HDL-C and elevated TGs reported in humans elsewhere [13,28]. Comparably, for resistin, the association with cardiometabolic risk seems to be moderated by body composition in line with previous literature [58]; nevertheless, previous studies also reported an association between resistin and other inflammatory cytokines, such as IL-6 and TNF-α, together with a diagnostic ability to predict human coronary atherosclerosis [63,64], which are the findings unconfirmed in the current study. 

Within the present study, cystatin-C consistently did not show any significant association with CM risk. This can be partially explained considering the previously reported lack of the specificity of the cystatin-C for the diagnostics, as a previous large cross-sectional study in the city of Groningen in the Netherlands demonstrated that other than renal function (which is the primary diagnostic purpose of this biomarker), a range of factors including age, gender, weight, height, smoking habits and the level of CRP were independently linked with serum cystatin-C since these associations remained significant even after adjusting for the creatinine clearance [124]. Similarly, another study conducted in the city of Nanjing, China, demonstrated that BMI, nephritis, kidney neoplasm and hypertension were the key confounding factors affecting cystatin-C amongst the older adults [125]. Despite this, we believe that our cystatin-C findings need to be treated with caution as several previous studies proposed the association between cystatin-C and MetS in premenopausal and postmenopausal women independent from age, BMI, lipid profile and lifestyle variables [126] in older adults [16] and in adults [91,94]. 

The present study piloted the feasibility of the use of biochip array technology for multiplex assessment of several circulatory metabolic markers. While some previous studies typically examined the association between one or a few biomarkers with MetS (e.g., [58,123,127,128,129,130]), the simultaneous analysis of several biomarkers in community dwelling older adults remains one of the strengths of the present study. This is important since a large part of the body of knowledge investigating the association between advanced circulatory metabolic markers and MetS include participants from hospitals and clinical settings (e.g., [58,127,131,132,133,134]) and often reports on associations potentially confounded by the presence of other pathologies. The focus on otherwise healthy community dwelling older adults can also be interpreted as a limitation of our study since, while it enhanced the internal validity of our work, the extensive list of exclusion criteria linked with pathologies may limit the broader generalizability of our findings.

Although we recruited healthy community-dwelling older adults, it is important to reflect on the high prevalence of hypertension within our sample. Hypertension is generally highly prevalent in older adults and the previous studies demonstrated that the USA and Europe prevalence ranged from 53% to 73% [135], while a more recent study from the National Health and Nutrition Examination Survey reported 70% of older American adults diagnosed with hypertension [136]. In England, a previous study reported only 34% of older adults with normal blood pressure (i.e., under 140/90 mmHG) [137]; however, direct comparison is challenging considering the criteria used for diagnosis in different studies; that national prevalence reports masks regional variations; the difference between reported community-dwelling diagnosed prevalence vs. doctor diagnosed; and the age groups and sampling used within our study. It is also important to consider that the criteria of hypertension used in NCEP-ATP III (130/85 mmHG) and, consequently, within our study is slightly different from some common clinical criteria used in other studies (140/90 mmHG) and our data included pre-hypertensive individuals in the prevalence.

The relatively small sample size (*n* = 100) of older adults who participated in this study is comparable with/larger than the sample size of several studies in the literature (e.g., [58,131,132,138,139]). We appreciate that, together with non-random sampling, this observation limited the external validity of the prevalence reported and believe this to be a key limitation adversely affecting the statistical power of our findings. Hence, we approach this limitation with full transparency, reflecting on very expensive, time-consuming and labor-intensive methods in our data collection and laboratory analysis of markers in free-living older adults and believe that our work can be used as a pilot/feasibility study for designing large scale cross-sectional studies and also for prioritizing the selection of biomarkers in future research and diagnostic/clinical settings.

## 5. Conclusions

The current study aimed to investigate the diagnostic power of a range of circulatory metabolic biomarkers as MetS risk correlates in community dwelling older adults in Northwest England. In almost all statistical analyses, fasting serum insulin concentration was statistically linked with MetS, its components and comorbidities. The statistically significant association between IL-6, TNF-α, resistin, CRP and CM risk was eliminated when multivariate OR from the logistic regression was adjusted for known confounding factors. The analysis of the area under ROC and precision-recall curve conducted for all potential circulatory biomarkers confirmed the discriminatory diagnostic power of insulin as MetS risk predictor with good AUC and AUPRC outcomes.

It is important to consider this study and its findings within the framework of the cross-sectional studies and their limitations to establish cause and effect relationships. While we investigated the associations between biomarkers and disease, it is unclear if the changed biomarkers are a cause, risk factor or the consequence of the syndrome and this remains an important and key question to be considered as part of the direction of the future research.

## Figures and Tables

**Table 1:** Definitions of Metabolic Syndrome [6,10].

	WHO	NCEP ATP III	IDF	EGIR	AHA
**Criteria**	IR or diabetes plus 2/5 of criteria below	Any 3/5 of criteria below	Obesity plus two of criteria below	Hyperinsulinemia plus two of the criteria below	≥3 of criteria below
**Obesity**	Waist/hip ratio >0.9 (M), >0.85 (W) or BMI > 30 kg/m^2^	WC ≥ 102 cm (M) ≥88 cm (W)	WC > 94 cm (M) >80 cm (W)	WC ≥ 94 cm (M) ≥80 cm (W)	WC ≥ 102 cm (M) ≥88 cm (W)
**Hyperglycemia**	IR or FBG > 7.8 mmol/L	FBG ≥ 5.6 mmol/L	FBG > 5.6 mmol/L	IR	FBG ≥ 5.6 mmol/L
**Elevated** **Triglycerides**	TGs > 1.7 mmol/L	TGs ≥ 1.7 mmol/L	TGs > 1.7 mmol/L	TGs > 2.0 mmol/L	TGs ≥ 1.7 mmol/L
**Decreased** **HDL-C**	<0.9 mmol/L (M), <1.0 mmol/L (W)	<1.0 mmol/L (M), <1.3 mmol/L (W)	<1 mmol/L (M), <1.3 mmol/L (W)	<1.0 mmol/L	<1.0 mmol/L (M), <1.3 mmol/L (W)
**Hypertension**	BP ≥ 140/90 mmHg or taking medication for hypertension	BP ≥ 130/85 mmHg	BP ≥ 130/85 mmHg	BP ≥ 140/90 mmHg or taking medication for hypertension	BP > 130/85 mmHg
**Other**	Urine albumin ≥20 μg/min or albumin creatinine ratio ≥30 mg/g				

Note: WHO: World Health Organization; NCEP-ATP III: National Cholesterol Education Program-Adult Treatment Panel III; IDF: International Diabetes Foundation; AHA: American Heart Association; EGIR: European Group for Study of Insulin Resistance; IR: Insulin Resistance; WC: Waist Circumference; M: Men; W: Women; BMI: Body Mass Index; BP: Blood Pressure; TGs: Triglycerides; HDL-C: High Density Lipoprotein Cholesterol.

**Table 2:** Characteristics of the study population.

Variable	Mean ± SE or %
Age (years)	68.73 ± 0.58
Sex (male: female) (%)	48:52
Height (cm)	166.51 ± 0.94
Weight (kg)	75.33 ± 1.65
BMI (kg/m^2^)	27.06 ± 0.52
Waist Circumference (cm)	90.19 ± 1.45
WHtR	0.54 ± 0.008
Body Fat	
*Low (%)*	4.2
*Optimal (%)*	41.1
*Moderate (%)*	34.7
*High (%)*	20
Skeletal Muscle Index	8.92 ± 0.11
Gait Speed	1.30 ± 0.02
Hand Grip	28.59 ± 0.89
Employed (%)	15
Married (%)	66
Live alone (%)	28
Education	
*Primary or Secondary (%)*	19
*Higher occupational (%)*	36
*University (%)*	45
SBP (mmHg)	143.92 ± 1.62
DBP (mmHg)	82.73 ± 1.17
MAP (mmHg)	103.13 ± 1.18
Pulse Wave Velocity	8.89 ± 0.22
HbA1c (%)	5.47 ± 0.04
Total Cholesterol (mmol/l)	5.18 ± 0.11
HDL-C (mmol/l)	1.56 ± 0.04
LDL-C (mmol/l)	2.97 ± 0.10
TGs (mmol/l)	1.44 ± 0.10
Fasting blood glucose (mmol/l)	5.51 ± 0.07
MetS prevalence	
*Prevalence based on NCEP ATP III definition (%)*	26
*Prevalence based on IDF definition (%)*	33
*Prevalence based on CMDS definition (%)*	22
Occurrence of individual MetS risk factors (as NCEP ATP III)	
*Elevated Waist Circumference (%)*	27
*Reduced HDL-C (%)*	13.5
*Elevated TGs (%)*	26.5
*Elevated fasting blood glucose (%)*	38.7
*Hypertension (%)*	88
*At least one risk factor of MetS present*	97
*At least two risk factors of MetS present*	59

Note: WHtR: Waist to Height Ratio; SBP: Systolic Blood Pressure; DBP: Diastolic Blood Pressure; HbA1c: Glycated Hemoglobin; HDL-C: High Density Lipoprotein Cholesterol; LDL-C: Low Density Lipoprotein Cholesterol; TGs: Triglycerides.

**Table 3:** Univariate linear regression of circulatory metabolic biomarkers with cardiometabolic risk parameters.

	Dependent	Blood Glucose (mmol/L)			LDL-C (mmol/L)			HDL-C (mmol/L)			TGs ^a^ (mmol/L)			HBA1C (%)		
Predictors		β	$r^{2}$	*p*-Value	β	$r^{2}$	*p*-Value	β	$r^{2}$	*p*-Value	β	$r^{2}$	*p*-Value	β	$r^{2}$	*p*-Value
IL-6 (pg/mL)		0.128	0.016	0.226	0.039	0.001	0.721	−0.122	0.015	0.246	0.094	0.009	0.370	0.083	0.007	0.422
Insulin (µlU/mL)		0.464	0.215	<0.0001 *	0.114	0.013	0.301	−0.457	0.208	<0.0001 *	0.361	0.130	<0.0001 *	0.201	0.040	0.055
TNF-α (pg/mL)		−0.045	0.002	0.668	0.182	0.033	0.089	−0.240	0.058	0.021	0.207	0.043	0.045	−0.115	0.013	0.268
Adiponectin (µg/mL)		−0.238	0.057	0.023	0.107	0.011	0.328	0.543	0.294	<0.0001 *	−0.124	0.015	0.239	−0.151	0.023	0.148
Leptin (ng/mL)		0.013	0.0002	0.899	0.028	0.001	0.795	−0.022	0.0005	0.835	0.166	0.028	0.110	−0.046	0.002	0.658
PAI-1 (ng/mL)		0.061	0.004	0.563	0.108	0.012	0.316	−0.163	0.027	0.120	0.265	0.070	0.010	0.071	0.005	0.492
Resistin (ng/mL)		0.203	0.041	0.053	−0.058	0.003	0.591	−0.167	0.028	0.111	0.078	0.006	0.457	0.011	0.0001	0.918
CRP (µg/mL)		0.194	0.038	0.065	−0.034	0.001	0.756	−0.071	0.005	0.500	0.169	0.029	0.103	0.160	0.026	0.121
Ferritin (ng/mL)		0.097	0.009	0.371	0.003	0.00001	0.982	−0.211	0.045	0.051	−0.125	0.016	0.247	0.045	0.002	0.673
Cystatin-C (µg/mL)		−0.054	0.003	0.612	0.146	0.021	0.174	0.069	0.005	0.514	0.106	0.011	0.308	0.012	0.0001	0.907
	**Dependent**	**WC** **(cm)**			**Weight** **(kg)**			**BMI** **(kg/m^2^)**			**Body Fat** **(%)**					
**Predictors**		**β**	$r^{2}$	***p*-Value**	**β**	$r^{2}$	***p*-Value**	**β**	$r^{2}$	***p*-Value**	**β**	$r^{2}$	***p*-Value**			
IL-6 (pg/mL)		0.309	0.095	0.002	0.298	0.089	0.003	0.374	0.140	<0.0001 *	0.386	0.149	<0.0001 *			
Insulin (µlU/mL)		0.550	0.303	<0.0001 *	0.574	0.330	<0.0001 *	0.462	0.214	<0.0001 *	0.283	0.080	0.008			
TNF-α (pg/mL)		0.094	0.009	0.364	0.125	0.016	0.224	0.094	0.009	0.360	0.099	0.010	0.350			
Adiponectin (µg/mL)		−0.396	0.157	<0.0001 *	−0.326	0.106	0.001	−0.189	0.036	0.069	0.105	0.011	0.327			
Leptin (ng/mL)		0.248	0.062	0.015	0.346	0.119	0.001	0.539	0.291	<0.0001 *	0.632	0.400	<0.0001 *			
PAI-1 (ng/mL)		0.153	0.024	0.136	0.186	0.035	0.069	0.148	0.022	0.151	0.180	0.033	0.087			
Resistin (ng/mL)		0.201	0.041	0.049	0.198	0.039	0.053	0.247	0.061	0.015	0.195	0.038	0.064			
CRP (µg/mL)		0.321	0.103	0.001	0.403	0.162	<0.0001 *	0.526	0.277	<0.0001 *	0.512	0.262	<0.0001 *			
Ferritin (ng/mL)		0.259	0.067	0.014	0.278	0.082	0.006	0.119	0.014	0.263	−0.090	0.008	0.411			
Cystatin-C (µg/mL)		−0.028	0.001	0.784	−0.006	0.00004	0.956	0.050	0.003	0.625	0.131	0.017	0.215			
	**Dependent**	**MAP** **(mmHG)**			**PWV** **(m/s)**			**AIP**			**HOMA1-IR ^a^**					
**Predictors**		**β**	$r^{2}$	***p*-Value**	**β**	$r^{2}$	***p*-Value**	**β**	$r^{2}$	***p*-Value**	**β**	$r^{2}$	***p*-Value**			
IL-6 (pg/mL)		0.224	0.050	0.030	0.261	0.068	0.012	0.124	0.015	0.241	0.281	0.079	0.007			
Insulin (µlU/mL)		0.129	0.017	0.221	0.173	0.030	0.102	0.509	0.259	<0.0001 *	0.934	0.873	<0.0001 *			
TNF-α (pg/mL)		−0.087	0.008	0.402	−0.009	0.0001	0.931	0.290	0.084	0.005	0.227	0.052	0.030			
Adiponectin (µg/mL)		0.006	0.00004	0.956	−0.075	0.006	0.483	−0.361	0.130	0.001	−0.474	0.225	<0.0001 *			
Leptin (ng/mL)		0.196	0.038	0.059	0.177	0.031	0.089	0.145	0.021	0.172	0.223	0.050	0.033			
PAI-1 (ng/mL)		−0.051	0.003	0.625	0.082	0.007	0.435	0.294	0.086	0.005	0.275	0.075	0.008			
Resistin (ng/mL)		0.011	0.0001	0.915	0.026	0.001	0.803	0.137	0.019	0.196	0.234	0.055	0.026			
CRP (µg/mL)		0.106	0.011	0.310	0.084	0.007	0.422	0.168	0.028	0.112	0.271	0.074	0.010			
Ferritin (ng/mL)		0.056	0.003	0.603	0.003	0.00001	0.976	−0.023	0.001	0.836	0.216	0.047	0.044			
Cystatin-C (µg/mL)		0.144	0.021	0.165	−0.007	0.00005	0.948	0.057	0.003	0.594	0.012	0.0001	0.914			

Note: ^a^ based on log transformation. * *p*-value < 0.0004 (Bonferroni correction was considered due to multiple comparison).

**Table 4:** Multivariate linear regression of circulatory metabolic biomarkers with cardiometabolic risk parameters.

	Dependent	Blood Glucose (mmol/L)		LDL-C (mmol/L)		HDL-C (mmol/L)		TGs ^a^ (mmol/L)		HBA1C (%)	
Predictors		β	*p*-Value	β	*p*-Value	β	*p*-Value	β	*p*-Value	β	*p*-Value
IL-6 (pg/mL)		0.054	0.688	−0.090	0.580	−0.020	0.877	−0.171	0.245	0.168	0.281
Insulin (µlU/mL)		0.489	<0.0001 *	−0.029	0.841	−0.278	0.016 *	0.335	0.011 *	0.199	0.148
TNF-α (pg/mL)		−0.308	0.016 *	0.244	0.134	−0.078	0.553	0.028	0.838	-0.261	0.075
Adiponectin (µg/mL)		−0.064	0.559	0.128	0.337	0.389	0.001 *	0.045	0.704	-0.130	0.302
Leptin (ng/mL)		−0.158	0.177	0.068	0.637	0.057	0.620	0.060	0.637	-0.201	0.139
PAI-1 (ng/mL)		0.014	0.899	0.172	0.214	0.007	0.952	0.302	0.018 *	0.124	0.350
Resistin (ng/mL)		0.283	0.012	−0.118	0.391	−0.002	0.987	−0.056	0.643	-0.016	0.904
CRP (µg/mL)		0.155	0.213	−0.057	0.710	0.056	0.652	0.021	0.880	0.066	0.649
Ferritin (ng/mL)		0.024	0.819	−0.005	0.967	−0.122	0.238	−0.259	0.025 *	0.007	0.952
Cystatin-C (µg/mL)		−0.018	0.862	0.148	0.239	0.073	0.474	0.121	0.281	0.050	0.671
**Adjusted-*r*^2^**		$r^{2}=0.281$		$r^{2}=0.009$		$r^{2}=0.313$		$r^{2}=0.142$		$r^{2}=0.025$	
***p*-value**		*p* < 0.0001 *		*p* = 0.396		*p* < 0.0001 *		*p* = 0.018 *		*p* = 0.299	
	**Dependent**	**WC** **(cm)**		**Weight** **(kg)**		**BMI** **(kg/m^2^)**		**Body Fat** **(%)**			
**Predictors**		**β**	***p*-Value**	**β**	***p*-Value**	**β**	***p*-Value**	**β**	***p*-Value**		
IL-6 (pg/mL)		0.145	0.236	0.011	0.923	−0.034	0.763	−0.050	0.667		
Insulin (µlU/mL)		0.454	<0.0001 *	0.485	<0.0001 *	0.361	<0.0001 *	0.118	0.247		
TNF-α (pg/mL)		−0.207	0.071	−0.173	0.118	−0.222	0.035 *	−0.179	0.093		
Adiponectin (µg/mL)		−0.238	0.017 *	−0.125	0.189	−0.012	0.898	0.161	0.094		
Leptin (ng/mL)		0.109	0.305	0.227	0.029 *	0.459	<0.0001 *	0.557	<0.0001 *		
PAI-1 (ng/mL)		−0.055	0.593	−0.037	0.711	−0.057	0.551	0.042	0.671		
Resistin (ng/mL)		0.066	0.515	0.029	0.770	0.139	0.135	0.176	0.065		
CRP (µg/mL)		0.051	0.654	0.153	0.165	0.272	0.010 *	0.261	0.016*		
Ferritin (ng/mL)		0.196	0.041 *	0.203	0.030 *	0.070	0.423	−0.060	0.497		
Cystatin-C (µg/mL)		−0.025	0.785	−0.009	0.919	0.041	0.631	0.124	0.162		
**Adjusted-*r*^2^**		$r^{2}=0.395$		$r^{2}=0.429$		$r^{2}=0.491$		$r^{2}=0.479$			
***p*-value**		*p* < 0.0001 *		*p* < 0.0001 *		*p* < 0.0001 *		*p* < 0.0001 *			
	**Dependent**	**MAP** **(mmHG)**		**PWV** **(m/s)**		**AIP**		**HOMA1-IR ^a^**			
**Predictors**		**β**	***p*-Value**	**β**	***p*-Value**	**β**	***p*-Value**	**β**	***p*-Value**		
IL-6 (pg/mL)		0.314	0.043 *	0.279	0.082	−0.172	0.214	0.063	0.241		
Insulin (µlU/mL)		0.239	0.078	0.208	0.138	0.409	0.001 *	0.896	<0.0001 *		
TNF-α (pg/mL)		−0.175	0.223	−0.092	0.532	0.086	0.524	−0.158	0.002 *		
Adiponectin (µg/mL)		−0.013	0.914	−0.030	0.813	−0.143	0.207	−0.111	0.013 *		
Leptin (ng/mL)		0.054	0.686	0.045	0.747	0.038	0.754	−0.017	0.712		
PAI-1 (ng/mL)		−0.121	0.354	0.026	0.844	0.230	0.051	0.096	0.036 *		
Resistin (ng/mL)		−0.015	0.904	0.002	0.988	−0.063	0.588	0.080	0.071		
CRP (µg/mL)		−0.109	0.445	−0.119	0.419	0.001	0.991	−0.039	0.433		
Ferritin (ng/mL)		0.035	0.772	−0.048	0.701	−0.177	0.099	−0.008	0.855		
Cystatin-C (µg/mL)		0.123	0.295	−0.003	0.978	0.074	0.484	0.030	0.465		
**Adjusted-*r*^2^** ***p*-value**		$r^{2}=0.064$ *p* = 0.138		$r^{2}=0.00006$ *p* = 0.450		$r^{2}=0.264$ *p* < 0.0001 *		$r^{2}=0.886$ *p* < 0.0001 *			

Note: ^a^ based on log transformation. * *p*-value < 0.05.

**Table 5:** Multivariate adjusted odds ratio (and 95% CI) for cardiometabolic risk associated with circulatory metabolic biomarkers.

	MetS		At Least Two Risk Factors	
	*Unadjusted OR (95%CI)*	*Adjusted OR (95%CI)*	*Unadjusted OR (95%CI)*	*Adjusted OR (95%CI)*
IL-6 (pg/mL)	1.32 (1.06–1.64)	1.21 (0.91–1.62)	1.34 (1.03–1.74)	1.35 (0.92–1.97)
Insulin (µlU/mL)	1.24 (1.12–1.37)	1.25 (1.09–1.43)	1.33 (1.16–1.53)	1.87 (1.24–2.83)
TNF-α (pg/mL)	1.37 (1.02–1.84)	1.22 (0.84–1.77)	1.14 (0.89–1.48)	1.38 (0.84–2.27)
Adiponectin (µg/mL)	0.90 (0.79–1.02)	0.91 (0.79–1.06)	0.94 (0.86–1.02)	0.89 (0.75–1.06)
Leptin (ng/mL)	1.04 (1.00–1.09)	1.002 (0.94–1.07)	1.08 (1.00–1.16)	1.03 (0.93–1.14)
PAI-1 (ng/mL)	1.06 (1.00–1.13)	1.05 (0.97–1.13)	1.04 (0.99–1.10)	1.04 (0.95–1.13)
Resistin (ng/mL)	1.27 (1.04–1.54)	1.20 (0.95–1.51)	1.37 (1.06–1.78)	1.44 (0.96–2.15)
CRP (µg/mL)	1.29 (1.09–1.54)	1.18 (0.96–1.46)	1.19 (0.97–1.46)	1.03 (0.76–1.41)
Ferritin (ng/mL)	1.003 (1–1.007)	1.003 (1–1.009)	1 (1.00–1.004)	1.00 (1–1.006)
Cystatin-C (µg/mL)	1.09 (0.73–1.61)	0.99 (0.57–1.74)	0.91 (0.62–1.36)	0.81 (0.45–1.44)

**Table 6:** Area Under Curve analysis for cardiometabolic risk associated with circulatory metabolic biomarkers.

	MetS		At Least Two Risk Factors	
	AUC (95%CI) ^a^	AUC ^b^	AUC (95%CI) ^a^	AUC ^b^
IL-6 (pg/mL)	0.629 (0.500–0.758)	0.644	0.613 (0.499–0.727)	0.629
Insulin (µlU/mL)	0.773 (0.653–0.893)	0.783	0.775 (0.683–0.866)	0.785
TNF-α (pg/mL)	0.615 (0.482–0.749)	0.635	0.518 (0.400–0.636)	0.563
Adiponectin (µg/mL)	0.620 (0.490 −0.749)	0.640	0.555 (0.435–0.675)	0.619
Leptin (ng/mL)	0.590 (0.447–0.734)	0.681	0.663 (0.552–0.775)	0.711
PAI-1 (ng/mL)	0.637 (0.520–0.755)	0.643	0.576 (0.455–0.698)	0.606
Resistin (ng/mL)	0.544 (0.394–0.694)	0.555	0.559 (0.444–0.673)	0.598
CRP (µg/mL)	0.593 (0.448–0.737)	0.620	0.614 (0.501–0.728)	0.678
Ferritin (ng/mL)	0.514 (0.382–0.646)	0.611	0.332 (0.219–0.446)	0.464
Cystatin-C (µg/mL)	0.447 (0.317–0.576)	0.744	0.429 (0.311–0.546)	0.377

Note: ^a^ Obtained by 5-fold cross-validation. ^b^ Obtained by bootstrapping.

**Table 7:** Diagnostic measures for cardiometabolic risk associated with circulatory metabolic biomarkers.

	5-Fold Cross-Validation									
	MetS					At Least Two Risk Factors				
	TPR	TNR	PPV	NPV	BA	TPR	TNR	PPV	NPV	BA
IL-6 (pg/mL)	16%	97.2%	66.7%	76.7%	56.6%	73.7%	25.6%	59.1%	40%	49.7%
Insulin (µlU/mL)	44%	94.4%	73.3%	82.7%	69.2%	71.9%	61.5%	73.2%	60%	66.7%
TNF-α (pg/mL)	0%	97.2%	0%	73.4%	48.6%	80.7%	5.1%	55.4%	15.4%	42.9%
Adiponectin (µg/mL)	0%	100%	0%	73.4%	50%	88.9%	25%	61.5%	62.5%	56.9%
Leptin (ng/mL)	4%	97.2%	33.3%	74.2%	50.6%	98.2%	7.7%	60.9%	75%	53%
PAI-1 (ng/mL)	0%	98.6%	0%	73.7%	49.3%	87.7%	15.4%	60.2%	46.1%	51.5%
Resistin (ng/mL)	16%	98.6%	80%	76.9%	57.3%	73.7%	25.6%	59.1%	40%	49.7%
CRP (µg/mL)	28%	97.2%	77.8%	79.3%	62.6%	94.6%	10%	59.5%	57.1%	52.3%
Ferritin (ng/mL)	0%	97%	0%	72.7	48.5%	94.3%	2.7%	58.1%	25%	48.5%
Cystatin-C (µg/mL)	4%	98.6%	50%	74.5%	51.3%	100%	7.5%	60.2%	100%	53.7%

Note: TPR: true positive rate or sensitivity, recall; TNR: true negative rate or specificity, selectivity; PPV: positive predictive value or precision; NPV: negative predictive value. BA: balanced accuracy.

## Data Availability

The data are available only to the journal editors and upon request from the corresponding author.

## Supplementary Materials

The following are available online at https://www.mdpi.com/article/10.3390/nu13072275/s1, Figure S1: Precision—Recall curve based on MetS as an outcome, Figure S2: Precision—Recall curve based on having at least two risk factors as an outcome. Table S1: Diagnostic parameters for cardiometabolic risk associated with circulatory metabolic biomarkers.
