# Supplementary material for: Diagnostic Power of Circulatory Metabolic Biomarkers as Metabolic Syndrome Risk Predictors in Community-Dwelling Older Adults in Northwest of England (A Feasibility Study)
Source: Nutrients. 2021 Jun 30;13(7):2275. doi: 10.3390/nu13072275 (PMC8308366; doi:10.3390/nu13072275)
Supplement: Supplementary file 1 [file nutrients-13-02275-s001.zip › nutrients-1248111- Suppl Figures S1&2.pdf]

Supplementary Figure S1: Precision- Recall curve based on MetS as an outcome.

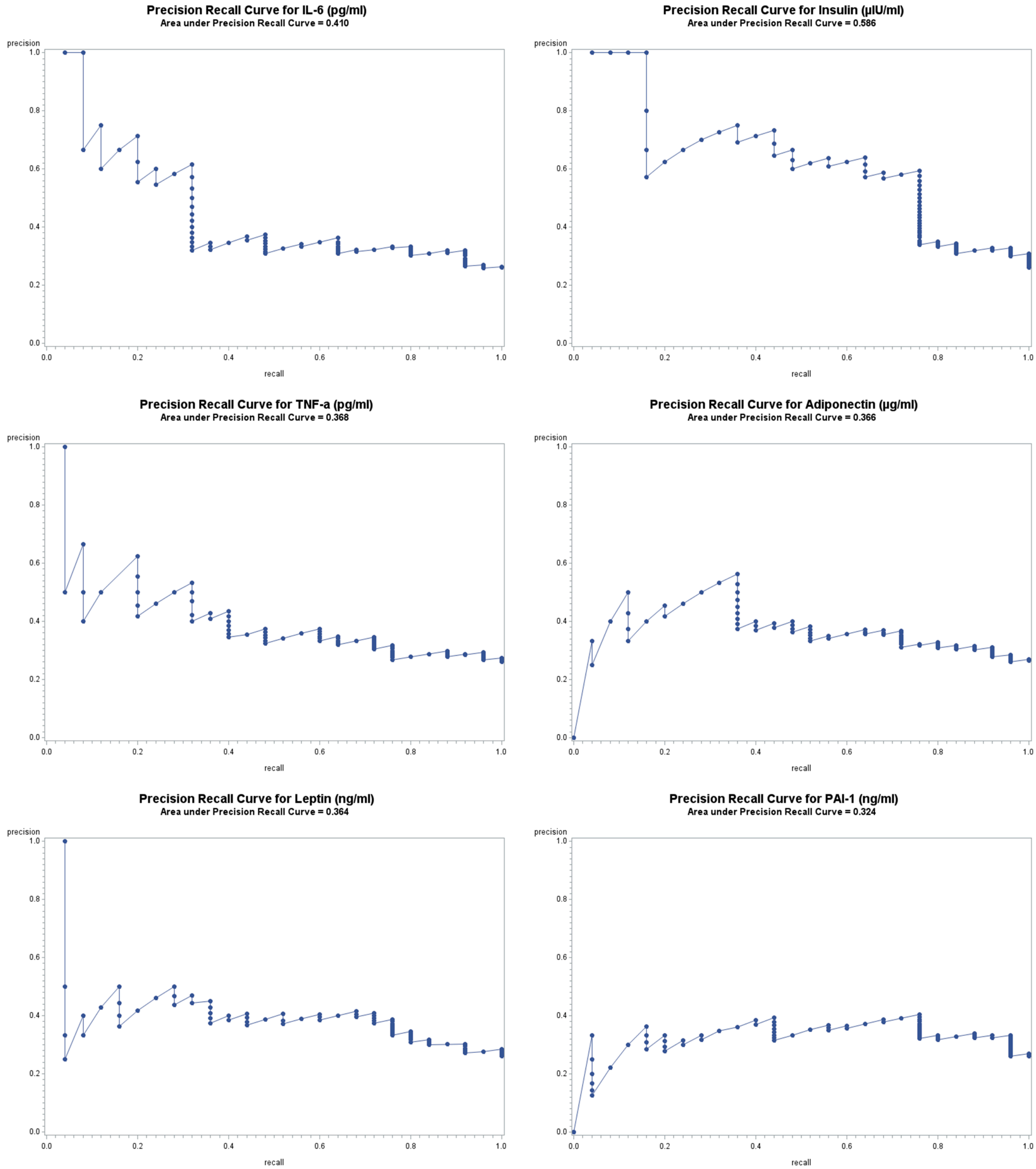

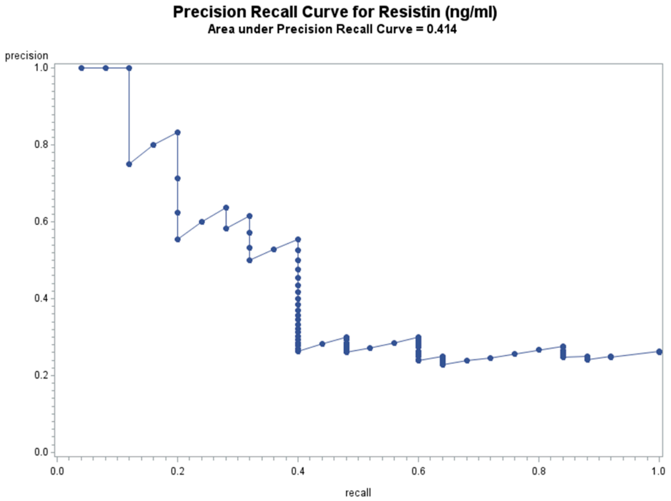
Precision Recall Curve for Resistin (ng/ml)
Area under Precision Recall Curve = 0.414
precision
1.0
0.8
0.6
0.4
0.2
0.0
0.0
0.2
0.4
0.6
0.8
1.0
recall

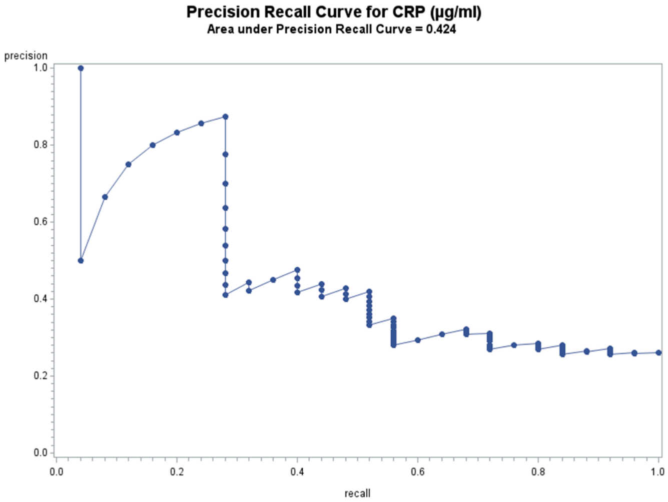
Precision Recall Curve for CRP (µg/ml)
Area under Precision Recall Curve = 0.424
precision
1.0
0.8
0.6
0.4
0.2
0.0
0.0
0.2
0.4
0.6
0.8
1.0
recall

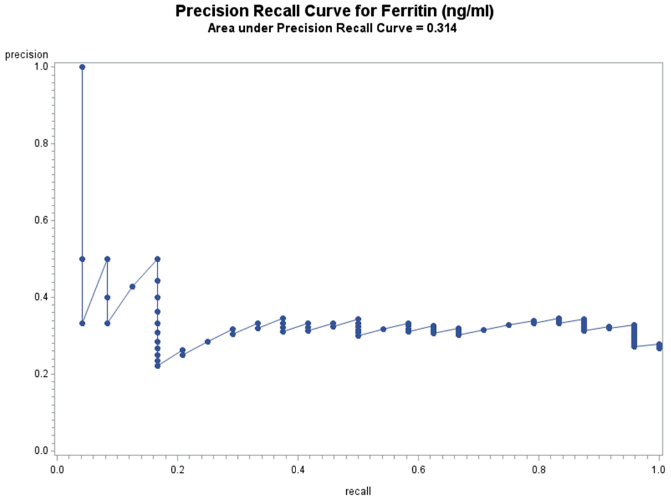
Precision Recall Curve for Ferritin (ng/ml)
Area under Precision Recall Curve = 0.314
precision
1.0
0.8
0.6
0.4
0.2
0.0
0.0
0.2
0.4
0.6
0.8
1.0
recall

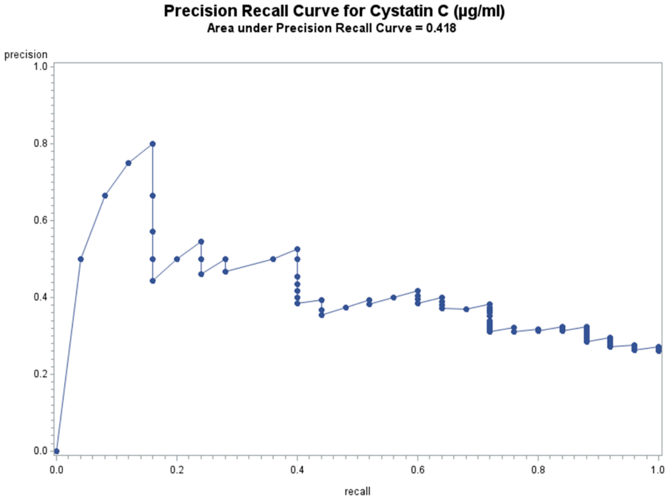
Precision Recall Curve for Cystatin C (µg/ml)
Area under Precision Recall Curve = 0.418
precision
1.0
0.8
0.6
0.4
0.2
0.0
0.0
0.2
0.4
0.6
0.8
1.0
recall

Supplementary Figure S2: Precision- Recall curve based on having at least two risk factors as an outcome.

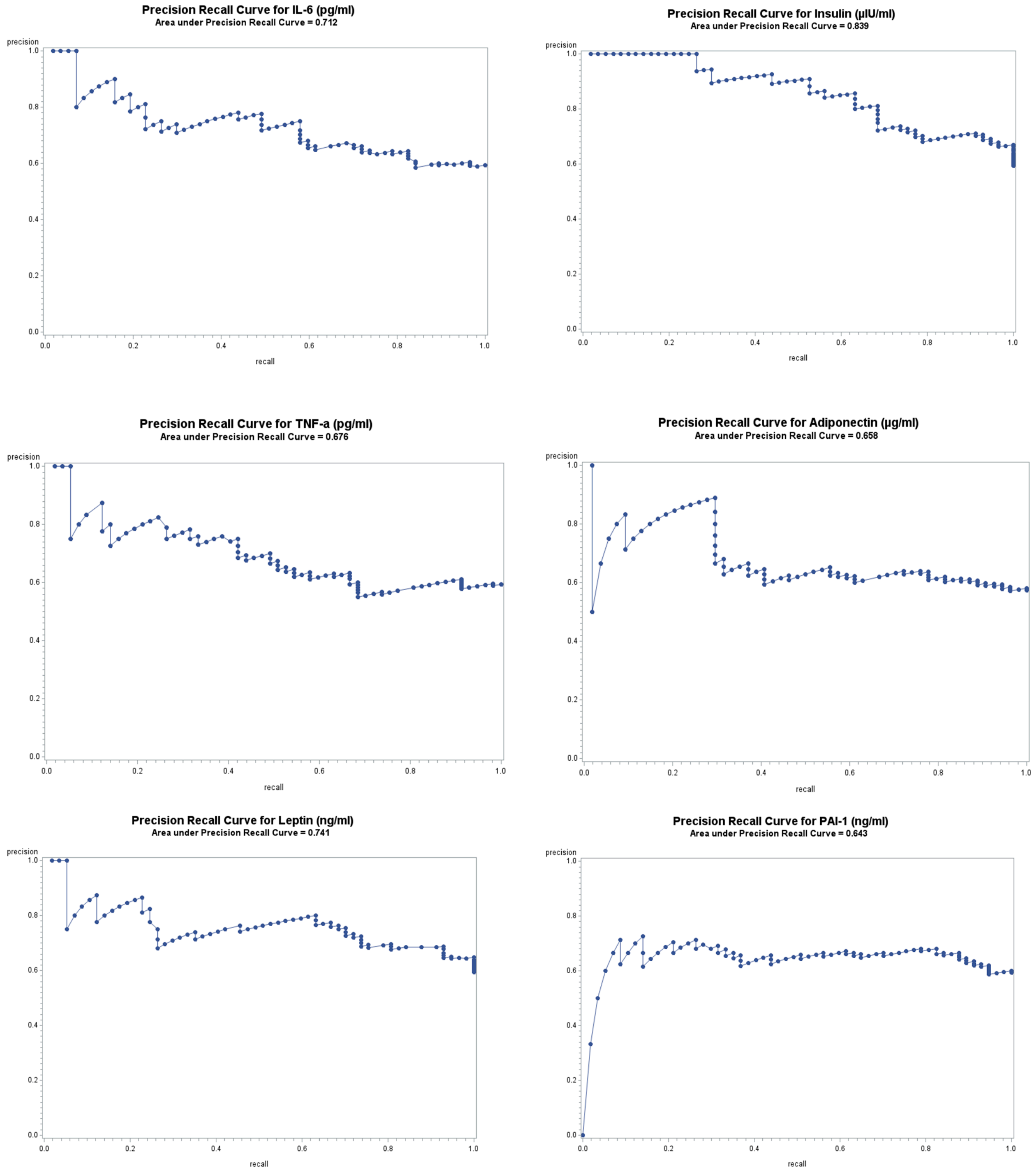

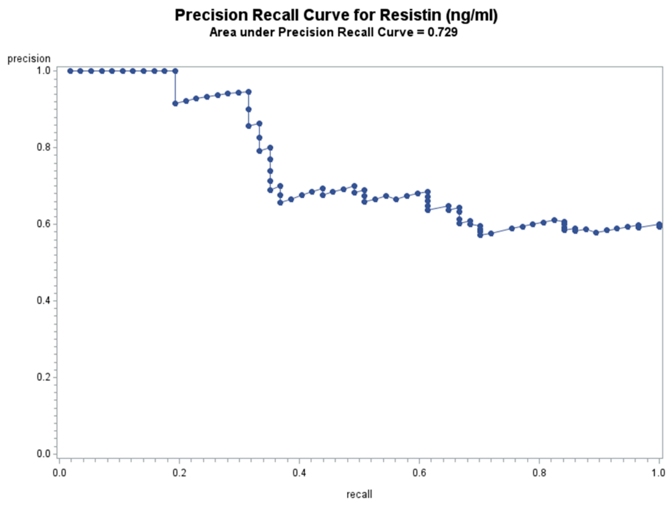
Precision Recall Curve for Resistin (ng/ml)
Area under Precision Recall Curve = 0.729
precision
recall

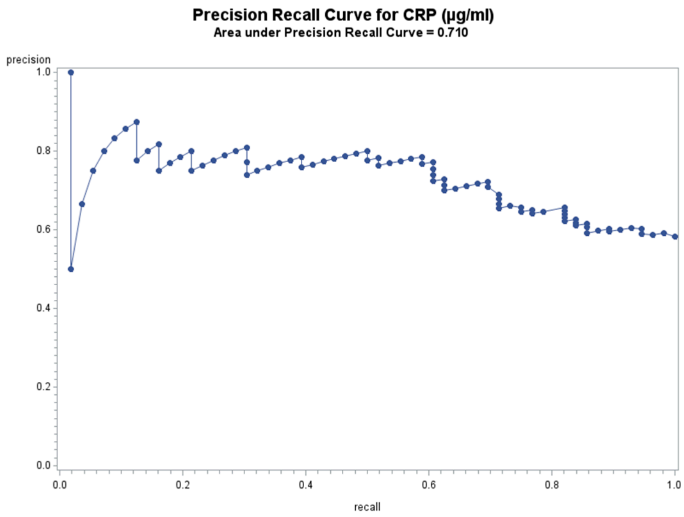
Precision Recall Curve for CRP (µg/ml)
Area under Precision Recall Curve = 0.710
precision
recall

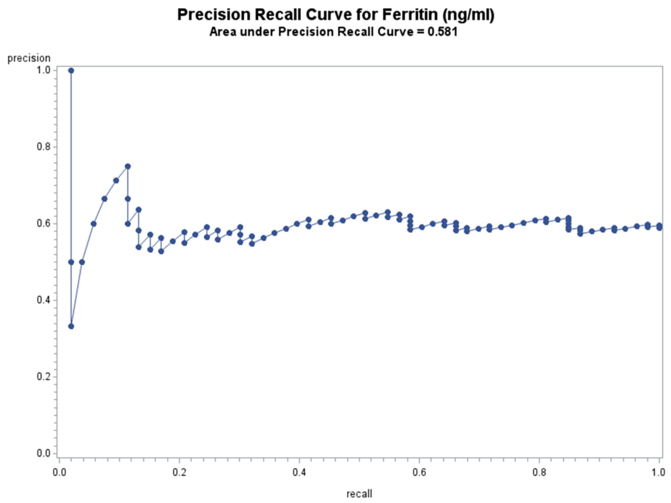
Precision Recall Curve for Ferritin (ng/ml)
Area under Precision Recall Curve = 0.581
precision
recall

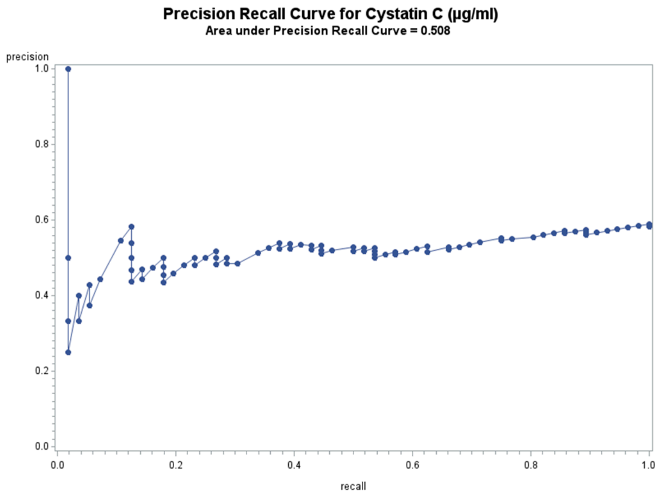
Precision Recall Curve for Cystatin C (µg/ml)
Area under Precision Recall Curve = 0.508
precision
recall
