# Supplementary material for: Diagnostic Power of Circulatory Metabolic Biomarkers as Metabolic Syndrome Risk Predictors in Community-Dwelling Older Adults in Northwest of England (A Feasibility Study)
Source: Nutrients. 2021 Jun 30;13(7):2275. doi: 10.3390/nu13072275 (PMC8308366; doi:10.3390/nu13072275)
Supplement: Supplementary file 1 [file nutrients-13-02275-s001.zip › nutrients-1248111- Suppl Table.pdf]

Supplemenatry Table S1. Diagnostic parameters for cardiometabolic risk associated with circulatory metabolic biomarkers.

	5-fold cross-validation									
	MetS					At least two risk factors				
	**TPR**	**TNR**	**PPV**	**NPV**	**BA**	**TPR**	**TNR**	**PPV**	**NPV**	**BA**
IL-6 (pg/ml)	16%	97.2%	66.7%	76.7%	56.6%	73.7%	25.6%	59.1%	40%	49.7%
Insulin (μlU/ml)	44%	94.4%	73.3%	82.7%	69.2%	71.9%	61.5%	73.2%	60%	66.7%
TNF-α (pg/ml)	0%	97.2%	0%	73.4%	48.6%	80.7%	5.1%	55.4%	15.4%	42.9%
Adiponectin (μg/ml)	0%	100%	0%	73.4%	50%	88.9%	25%	61.5%	62.5%	56.9%
Leptin (ng/ml)	4%	97.2%	33.3%	74.2%	50.6%	98.2%	7.7%	60.9%	75%	53%
PAI-1 (ng/ml)	0%	98.6%	0%	73.7%	49.3%	87.7%	15.4%	60.2%	46.1%	51.5%
Resistin (ng/ml)	16%	98.6%	80%	76.9%	57.3%	73.7%	25.6%	59.1%	40%	49.7%
CRP (μg/ml)	28%	97.2%	77.8%	79.3%	62.6%	94.6%	10%	59.5%	57.1%	52.3%
Ferritin (ng/ml)	0%	97%	0%	72.7	48.5%	94.3%	2.7%	58.1%	25%	48.5%
Cystatin C (μg/ml)	4%	98.6%	50%	74.5%	51.3%	100%	7.5%	60.2%	100%	53.7%
	**Bootstrapping**									
	**MetS**					**At least two risk factors**				
	**TPR**	**TNR**	**PPV**	**NPV**	**BA**	**TPR**	**TNR**	**PPV**	**NPV**	**BA**
IL-6 (pg/ml)	17.8%	96.4%	63.4%	76.9%	57.1%	82.2%	27.1%	62.2%	51%	54.6%
Insulin (μlU/ml)	41.6%	93.8%	70.4%	82%	67.7%	73.3%	62.3%	74%	61.5%	67.8%
TNF-α (pg/ml)	7.8%	97.2%	50.1%	75%	52.5%	87.5%	12.4%	59.3%	40.5%	50%
Adiponectin (μg/ml)	2%	99.1%	44.6%	73.6%	50.5%	86.3%	20.7%	59.5%	52.8%	53.5%
Leptin (ng/ml)	8%	96.3%	43.3%	74.8%	52.1%	86.3%	27.1%	63.4%	57.6%	56.7%
PAI-1 (ng/ml)	4.1%	95.9%	26.1%	74%	50%	87.5%	21.2%	61.9%	53.7%	54.3%
Resistin (ng/ml)	15.8%	98.2%	75.2%	76.8%	57%	74.1%	32.6%	61.7%	46.3%	53.4%
CRP (μg/ml)	27.2%	97.8%	81.5%	79.2%	62.5%	0.83.5%	27%	61.6%	53.9%	55.2%
Ferritin (ng/ml)	4.2%	97.7%	39.6%	73.7%	50.9%	89.7%	10.6%	59%	41.8%	50.2%
Cystatin C (μg/ml)	7.3%	97.8%	54%	75%	52.5%	88.7%	16.1%	59.1%	46.9%	52.4%

TPR: true positive rate or sensitivity, recall.
TNR: true negative rate or specificity, selectivity.
PPV: positive predictive value or precision.
NPV: negative predictive value.
BA: balanced accuracy.
